# Tyrosine-derived stimuli responsive, fluorescent amino acids†
†Electronic supplementary information (ESI) available: Full experimental details and characterization data of all the new compounds. See DOI: 10.1039/c4sc02753a



**DOI:** 10.1039/c4sc02753a

**Published:** 2014-10-31

**Authors:** Pradeep Cheruku, Jen-Huang Huang, Hung-Ju Yen, Rashi S. Iyer, Kirk D. Rector, Jennifer S. Martinez, Hsing-Lin Wang

**Affiliations:** a C-PCS, Chemistry Division , Los Alamos National Laboratory , Los Alamos , New Mexico 87545 , USA . Email: hwang@lanl.gov; b Defense System and Analysis Division , Los Alamos National Laboratory , Los Alamos , New Mexico 87545 , USA; c Center of Integrated Nanotechnologies (CINT) , Los Alamos National Laboratory , Los Alamos , New Mexico 87545 , USA

## Abstract

A series of fluorescent unnatural amino acids (UAAs) bearing stilbene and *meta*-phenylenevinylene (*m*-PPV) backbone have been synthesized and their optical properties were studied. These novel UAAs were derived from protected diiodo-l-tyrosine using palladium-catalyzed Heck couplings with a series of styrene analogs. Unlike the other fluorescent UAAs, whose emissions are restricted to a narrow range of wavelengths, these new amino acids display the emission peaks at broad range wavelengths (from 400–800 nm); including NIR with QY of 4% in HEPES buffer. The incorporation of both pyridine and phenol functional groups leads to distinct red, green, and blue (RGB) emission, in its basic, acidic and neutral states, respectively. More importantly, these amino acids showed reversible pH and redox response showing their promise as stimuli responsive fluorescent probes. To further demonstrate the utility of these UAAs in peptide synthesis, one of the amino acids was incorporated into a cell penetrating peptide (CPP) sequence through standard solid phase peptide synthesis. Resultant CPP was treated with two different cell lines and the internalization was monitored by confocal fluorescence microscopy.

## Abstract 2

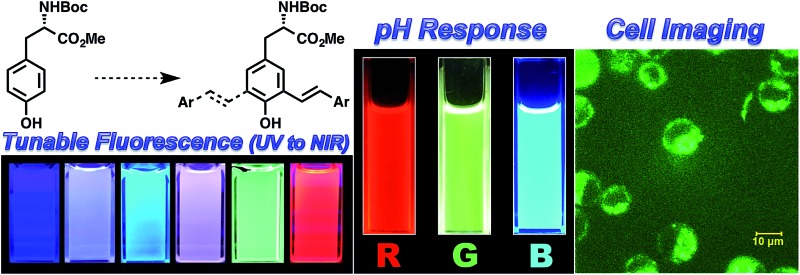
A series of fluorescent unnatural amino acids (UAAs) bearing stilbene and *meta*-phenylenevinylene (*m*-PPV) backbone have been synthesized by palladium-catalyzed Heck couplings.

## 1. Introduction

Fluorescence spectroscopy emerged as a powerful tool to investigate complex biological processes such as enzyme activity, protein structure and their interaction with other proteins and nucleic acids.^1^ Among the other natural amino acids, tryptophan and tyrosine serve as intrinsic fluorescent probes^2^ to monitor the above-mentioned processes but suffer from poor optical properties. Therefore continuous efforts have been made to design unnatural amino acids (UAAs) with various fluorophores and successfully incorporated into peptides and proteins in a site-specific manner to study their structure–function properties.^3^ Furthermore, relatively smaller size and ease of synthesis of UAAs, as compared to their fluorescent protein cohorts, allows chemists fine-tune the structure to obtain desired optical properties. Besides, UAAs bearing functionalities are stimuli-responsive serve as fluorescent reporters of micro-environmental changes such as pH, polarity, and redox.^4^

Owing to the advancement in synthetic biology methods and imaging techniques, there is always an imperative need for enrichment of UAAs' toolkit encompassing a variety of fluorescent scaffolds with diverse spectroscopic properties, shapes and sizes. As the assimilation of synthetic chemistry, biology and imaging furthers, the development of novel fluorescent UAAs will continue to be at the forefront to aid the researchers in understanding the fundamental yet complicated biological functions such as protein interactions, recognition, and biosynthesis.

From the synthetic chemistry point of view; two common approaches can be adopted to synthesize the fluorescent probes in the form of UAAs, (i) integrating known fluorophores into the side-chains of α-amino acids.^5*a*^ For example, a polarity-sensitive fluorescent UAA, L-Anap was synthesized *via* covalent attachment of naphthyl fluorophore to the hydroxyl group of l-serine using Fukuyama–Mitsunobu reaction,^5*b*^ and (ii) constructing a whole new chromophore on a natural amino acid. For example, coumarin-bearing fluorescent UAAs were derived from aspartic and glutamic acids;^6^ 2-(2-furyl)-3-hydroxychromone to probe peptide–nucleic acid complexes was synthesized from l-tyrosine.^7^ Latter approach has the advantage of being relatively nonperturbing replacements for the native residues, thereby maintaining the overall native structure of a target peptide or protein.^1*a*^ Additionally, a higher chemical stability is expected if the fluorophore is linked to the amino acids by a side-chain carbon–carbon bond.

**Fig. 1:**
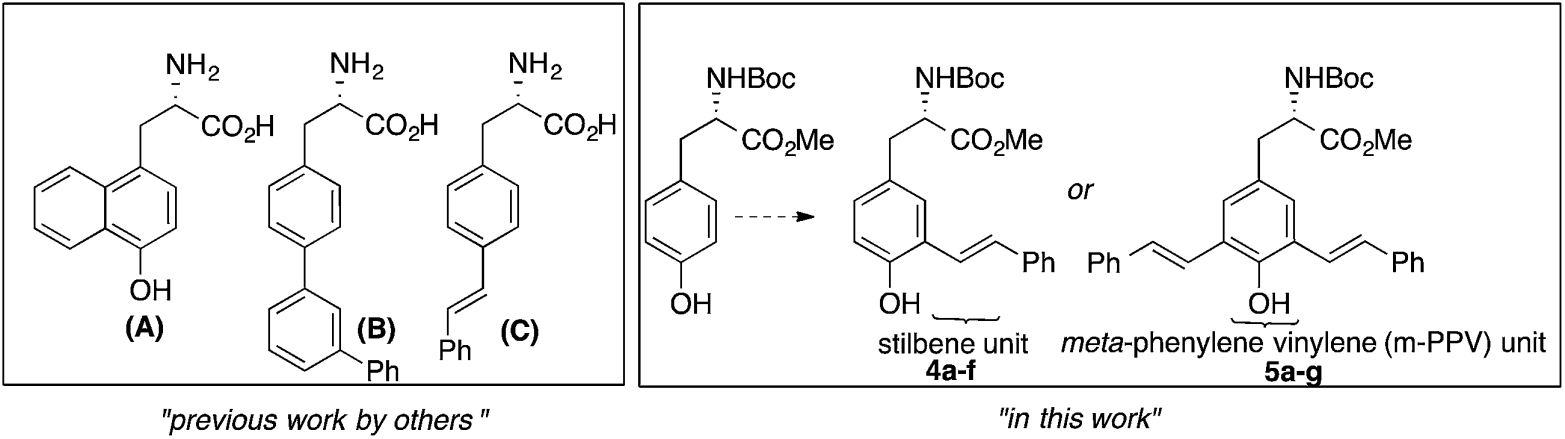
Fluorescent UAAs derived from tyrosine/phenylalanine by extending the π-conjugation of aromatic side chain.

As shown in Fig. 1, augmenting the π-conjugation in tyrosine/phenylalanine to generate structurally novel fluorescent UAAs is a unique approach. These UAAs (**A–C**) showed improved optical properties than the corresponding tyrosine/phenylalanine amino acids but suffered from multistep synthetic routes and lack of tunability in emission property.^8^

Recently, π-conjugated organic systems have received a lot of interests owing to their tunable physical, optical and electronic properties through tailored synthesis of conjugated structures with backbone and/or side chains that renders desired properties.^9^ Due to their planar and semi-rigid backbone and π–π stacking potential, these molecules have the propensity to form aggregates that show distinct electronic and optical properties. The physical and optical properties of conjugated molecules can also be fine-tuned by varying the nature of the substitution groups on the terminal phenyl rings.^10^ In addition to application in electronic, optical and energy devices, conjugated oligomers are also extensively used in biological and medicinal chemistry.^11^ For example, the use of styrene-based compounds as imaging agents and inhibitors of beta amyloid fibrils is well documented. Recently, Anna and co-workers reported on the synthesis of thiophene-based conjugated oligomers bearing l-amino acid and their use as optical probes for detection of amyloid fibril formation in insulin.^12^

Encouraged by recent developments in fluorescent UAAs, we envisage that the transformation of amino acids into conjugated systems would lead to a whole new class of fluorescent UAAs with desired optical properties (Fig. 1). In this regard, herein, we report the design and synthesis of novel α-amino acid analogs constructed *via* extending the π-conjugation of l-tyrosine. These novel fluorescent UAAs consist of stilbene and *meta*-phenylene vinylene units as fluorophores and cover the emission color from blue to near IR. Another unique structural feature is the incorporation of hydroxyl group (phenol) that renders stimuli responsive optical properties. More interestingly, we have observed distinct red, green and blue (RGB) emission spectra simply by controlling the solution pH. We also show the use of these UAAs in solid-phase peptide synthesis (SPPS) to synthesize a cell-penetrating peptide and demonstrate the use of these fluorescent peptides for cell imaging.

## 2. Results and discussion

### Synthesis of π-conjugation extended l-tyrosine amino acids

**Scheme 1:**
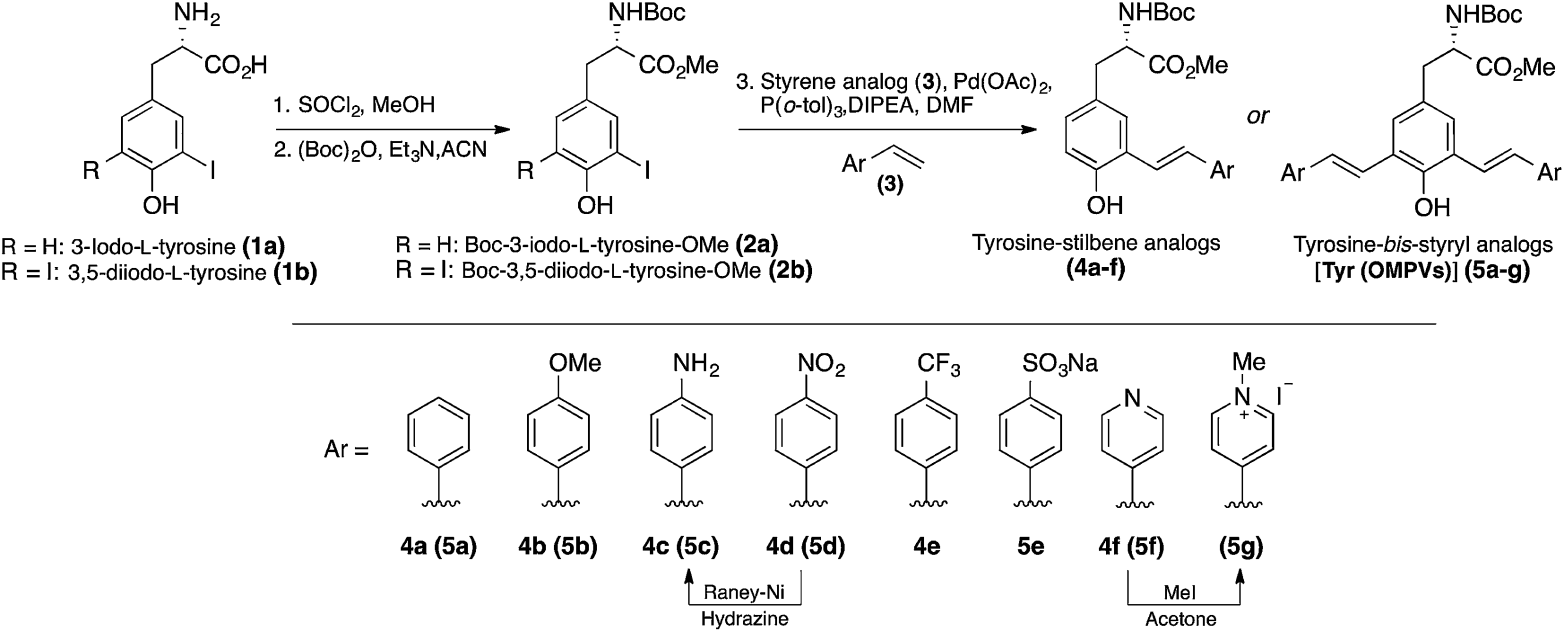
Synthesis of fluorescent unnatural amino acids **4a–f** and **5a–g** starting from tyrosine.

The *mono*- and *bis*-styryl-l-tyrosine analogs (compounds **4a–f** and **5a–g**) were synthesized using strategies shown in Scheme 1. The syntheses of these amino acids have begun with the transformation of commercially available 3-iodo-l-tyrosine (**1a**) and 3,5-diiodo-l-tyrosine (**1b**) into their corresponding *t*-Boc/OMe protected amino acids (**2a** and **2b**, respectively) by following the established literature procedures. Starting from natural amino acid is particularly advantageous because of two reasons:^1^ avoiding expensive chiral auxiliaries, which are usually required for the diastereoselective synthesis and thus minimizing the number of steps in synthesis,^13^ and^2^ the ability to secure the chiral information at the beginning of the synthesis.^14^ Although *bis*-styryl conjugated compounds can be obtained by a variety of synthetic pathways, the use of Heck reactions to synthesize *E*-configured styryl compounds has been proved to be most promising.^15^

Palladium catalyzed mono/double Heck couplings between **2a**/**2b** and appropriate styrene (**3a–f**) afforded protected tyrosine analogs **4a–f** and **5a–g**, bearing *meta*-phenylenevinylene backbone in moderate to good yields. Mindful selection of styrene precursors with different electron-withdrawing and electron-donating groups gave an access to thirteen fluorescent UAAs with different end groups, which allowed us to better understand the interplay between dipole interactions and aggregate formation, and fully assess their impact on the electronic and optical properties of π-conjugated UAAs. It is noteworthy that all the Heck couplings proceeded smoothly even without protecting the phenol group, thus minimizing the number of steps in synthesis. Reaction times for the Heck couplings depend on the substituents on the styrene compounds. In general, the presence of electron withdrawing groups at *para*-position of the styrene analog requires longer reaction times with slightly decreased yields.

We observed that all the Heck couplings progressed with sufficiently high selectivity to generate *E*-isomers as established by the NMR spectra. The *trans*-relation of the double bonds was established on the basis of the coupling constant for the vinylic protons in the ^1^H NMR spectra (*J* ∼ 16 Hz, ESI†).

All of these amino acids are stable as solids at room temperature and can be stored without the need of any special precautions. Absorption and fluorescence emission, extinction coefficients, fluorescence quantum yields (QY), and optical rotations were measured for each compound and presented in Table 1. The optical properties of l-tyrosine were included for comparison.^16^

**Table 1:** Physicochemical properties of tyrosine derived fluorescent amino acids^*a*^

Analog	*λ*_Abs_ (nm)	*λ*_Em_ (nm)	*ε* ^*b*^ [cm^–1^ M^–1^]	QY^*c*^	[*α*]25D^*d*^
**4a**	300, *345*	400	11 000	0.41	+15
**4b**	292, *335*	400	16 000	0.87	+16
**4c**	320, *360*	440	14 000	0.13	+11
**4d**	298, *390*	—	18 000	—	+10
**4e**	300, *340*	440	25 000	0.12	+15
**4f**	300, *340*	440	19 000	0.13	+12
**5a**	300, *355*	430, 512, 580	26 000	0.51	+ 8
**5b**	300, *360*	420, 438	29 000	0.94	+10
**5c**	320, *375*	490	30 000	0.47	+ 8
**5d**	400, *660*	–(0.11)^*e*^	39 400	–(0.08)^*e*^	+12
**5e**	*300*	410, 595	28 000	0.32	+15
**5f**	300, *370*, 520	630	34 000	0.24	+12
**5g**	370, *520*	800	28 000	0.002 (0.04)^*f*^	+10
Tyr	278	352	5300	0.12	—

^*a*^Determined in DMSO (*c* = 10 uM).

^*b*^Extinction coefficient.

^*c*^Quantum yield using 9,10-diphenyl anthracene as a standard reference.

^*d*^See ESI.

^*e*^QY in THF.

^*f*^QY in HEPES buffer (pH = 7.3); italics: excitation wavelengths.

### UV/vis and fluorescence spectroscopy

**Fig. 2:**
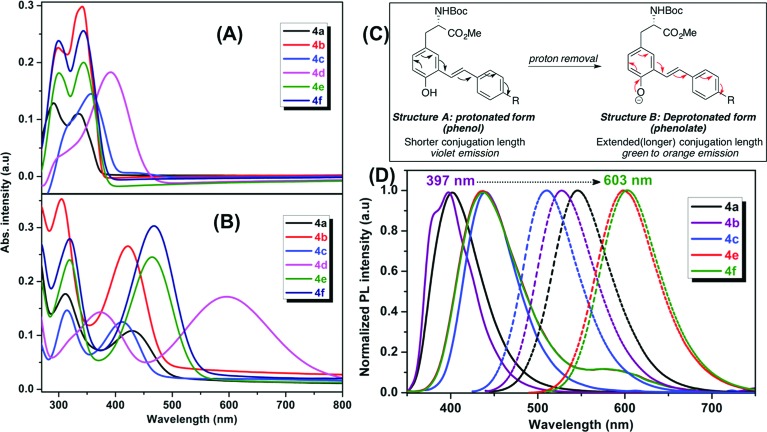
Absorption and emission spectra of mono-styryl tyrosine analogs **4a–f** in DMSO: UV-vis spectra (A) before the addition (B) after the addition of NaOH; (C) a clear red-shift in emission spectra is due to the extended conjugation of the phenolate ion; (D) PL spectra; before (solid line) and after (dotted lines) the addition of NaOH.

**Fig. 3:**
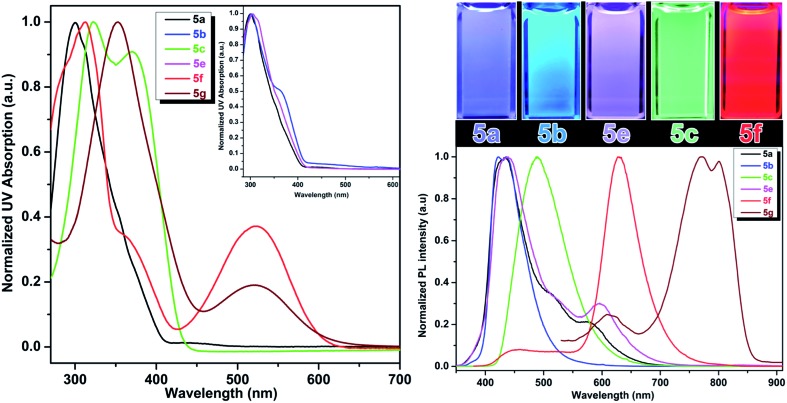
Absorption (left) and emission (right) properties of compounds **5a–f** in DMSO; left inset: overlapped absorption spectra of **5a**, **5b**, **5e**; right inset: emission colors of fluorescent amino acids in DMSO that covers the visible spectrum from violet to red.

The optical properties of the fluorescent amino acids, **4a–f** and **5a–g** were measured by UV/vis and fluorescence spectroscopy in DMSO at room temperature. The results are shown in Fig. 2 and 3.

### Mono-styryl-l-tyrosine analogs

We observed two characteristic absorption peaks in the UV-vis spectra of all *mono*-styryl analogs demonstrating the distinct electronic transitions. For example, all six amino acids have a higher energy absorption band with a *λ*_max_ at roughly 300 nm. For the low energy absorption peak, *λ*_max_ varies from 330 nm to 390 nm depending upon the nature of the *para*-substitution on the styrene ring.

For all these analogs, photoexcitation at the wavelength that corresponds to both low and high energy peaks show the same emission maxima, but the excitation of the low-energy peak resulted in higher emission intensities. We observed that the DMSO solutions of all amino acids in this series emitted strongly in violet region (400 nm to 435 nm), regardless the nature of substitution group on the styrene ring. The NO_2_ compound **4d** found to be nonemissive in highly polar (both protic and aprotic) solvents, such as DMSO, methanol, where as it is emissive in non-polar solvents such as chloroform and THF with an emission maxima 400 nm. Compounds **4e–f** having electron withdrawing groups (CF_3_ and Py) showed a weak shoulder band in emission spectrum at around 590 nm.

Our *mono*-styrene analogs emit at higher wavelengths (violet-blue) due to their short π-conjugation lengths is consistent with literature observation.^17^ As illustrated in Fig. 2, these amino acids are derived from tyrosine and composed of a phenol moiety that functions as a latent donor. Deprotonation of phenol leads to the formation of a phenolate active donor that donates a pair of π-electrons into the π-system and thus forming an extended π-conjugated system. As a result of this, a red shift in the emission of these amino acids was observed with increased emission intensities. Moreover, deprotonated phenol also gives rise to a stronger dipole, which facilitates the red shift of emission spectra in a polar solvent. Addition of a base to the DMSO solutions of these amino acids resulted in a red shift in their emissions pushing the emission maxima from 400–435 nm (violet) nm to 510–600 nm (green-orange). This result is in agreement with generation of the phenolate anion, while the spectra of phenol-protected derivatives unaltered by addition of base (ESI†).

### Bis-styryl-l-tyrosine analogs (Tyr-OMPVs)

Addition of another phenylene vinylene to the *meta* position of stilbene derivatives leads to a new class of *meta*-phenylene vinylene bearing amino acids (**5a–g**). All these amino acids showed absorption wavelengths (*λ*_max_) in the UV or visible region ranging from 300–430 nm (Fig. 3). In some cases, multiple absorption bands of lower energies were observed. For example, compounds **5d**, **5f** and **5g** showed the absorption peaks at lower energies (620 nm and 520 nm respectively, Fig. 3). The emergence of the low energy peak is likely due to the donor–acceptor characteristic of the molecules, which causes the HOMO and LUMO to merge, producing smaller band gap energy.^10^

As shown in Fig. 3, upon photoexcitation at a wavelength that corresponds to the absorption maxima, all these compounds showed a strong emission in the visible region encompassing the whole visible spectrum (from 400–800 nm), is a key feature of these amino acids. Compounds **5a** and **5e**, possessing H and SO_3_Na groups, respectively, showed emission in the blue region. Emission spectra of these compounds also have a small shoulder near 600 nm. As **5a** and **5e** showed a broad emission starting from 350 nm to 700 nm; these compounds looked more whitish when visualized under UV lamp. Compound **5b** and **5c** possessing electron-donating groups such as OMe and NH_2_, displayed blue and green emission at 420 nm and 500 nm, respectively. When compared to the other analogs in this series, compound **5d**, bearing NO_2_ groups on *para*-positions of the terminal phenyl rings, was not emissive in polar solvents such as DMSO and methanol. However, in apolar solvents such as THF and chloroform, it was emissive with a *λ*_max_ at 512 nm. **5d** in polar solvents is nonemissive which is attributed to a complex interplay of single molecule and aggregate emission observed for this particular NO_2_ containing compounds. **5d** aggregates in DMSO have been detected by dynamic light scattering (DLS) which shows a bimodal distribution with aggregate size of ∼100 nm. While in THF, compound **5d** is mostly monodispersed, non-aggregated species (Fig. S1 in ESI†).

The quantum yields of these UAAs in DMSO range from 94% to 4% depending on the solubility and functional groups attached to the end of the styryl part. A red shift of the emission bands was observed when the terminal phenyl rings were replaced with pyridine rings. Compound **5f** showed emission in the red region (630 nm) and this emission behavior could be due to the combined effect of the deprotonation of phenol and formation of aggregates. The aggregation behavior of **5f** is further validated by concentration dependent fluorescence experiments as well as DLS experiments. The aggregate-associated change in PL was confirmed by the concentration experiments, in which we measure PL spectra of **5f** in DMSO at concentrations ranging from 1 mM to 100 nM concentrations (Fig. S2, ESI†). At concentrations above 100 nM, the PL emission is dominated by with a *λ*_max_ at 630 nm and a small shoulder band at 430 nm. But, at 100 nM and lower concentrations, we observed that the peak at 630 nm was almost disappeared and the emission peak at 430 nm became dominant. The above results suggest that the blue emission at 430 nm and the red emission at 630 nm are associated with a single molecule and aggregate emission, respectively. It is very important to note that although aggregation induced red shift in fluorescence emission with low quantum yield is a well-known phenomenon in conjugated polymers/oligomers,^18^ **5f** aggregates actually has a fairly high quantum yield comparable to that of its single molecule species. We believe this is probably due to the formation of linear aggregates through H-bonding interaction, rather than π–π interaction between phenyl rings, which typically leads to the quenching of fluorescence.

On the other hand, compound **5f** showed the blue emission in apolar solvents such as THF (Fig. S3, ESI†) and chloroform, suggesting that these solvents do not facilitate the formation of aggregation as the blue emission is solely coming from the single molecule/non-aggregated species. Compound **5f** aggregates have been detected by dynamic light scattering (DLS) with the size of ∼70 nm in DMSO. While under diluted concentrations (<100 nM) in DMSO and in THF, no noticeable aggregate formation was observed. This result is in agreement with the concentration dependent fluorescence spectra, which suggest predominant single molecule species at lower concentrations (<100 nM).

### Stimuli response

#### Red, green, blue (RGB) emission

It has been shown that molecules comprise of nitrogen containing heterocyclic rings such as pyrimidines showed the ability to function as colorimetric and luminescent pH sensors due to the basic character of the nitrogen atoms of the pyrimidine ring.^19^ Such character prompts us to study the effect of protonation/deprotonation on the optical properties.

**Fig. 4:**
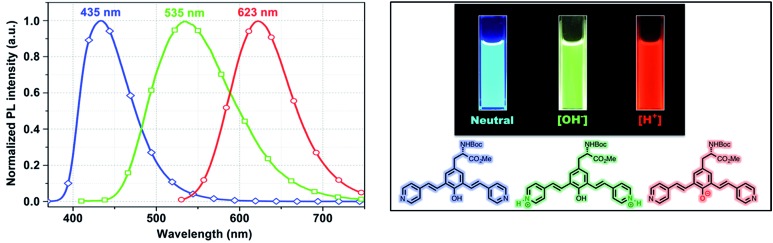
Emission spectra of **5f** in neutral (blue), acidic (green) and basic (red) environment showing the RGB emission.

One of the very interesting structural characteristics of **5f** is that it contains basic nitrogen atoms that can be protonated and a phenol group that can be deprotonated. Thus, the effect of protonation/deprotonation on the optical properties was found to be particularly interesting. In acetonitrile, **5f** underwent a significant visible color change upon the addition of acid *p*TSA (*p*-toluenesulfonic acid) or base (NaOH). Basic, acidic, and neutral solutions of **5f** showed red green and blue (RGB) emission respectively, see Fig. 4. Visible/emission color change is fully reversible by neutralization with an acid/base. The emission spectra in acid and basic solutions show a clear red shift when compared to the neutral solution. Emission peak at 535 nm corresponds to the protonated species whereas; the peak at 630 nm represents the phenolate structure.

As expected, most of the compounds exhibit a red shift of their absorption (Fig. S4, ESI†) and emission bands upon addition of *p*TSA and can be explained by the protonation enhances the accepting effect of the pyridine, thus increasing intramolecular charge transfer from the donor to the pyridinium moiety (acceptor).^20^ For the protonated species, the emission is partially quenched (QY = 10%) when compared to its neutral state (QY = 45%). Conversely, addition of NaOH enriched the phenolate population and as a result of extended conjugation, compound **5f** exhibited a more intense (QY = 57%) and red shifted emission at 630 nm. UAA with pH dependent fluorescence emission exhibits distinct red, green and blue color (RGB) with decent quantum yield suggests implications toward sensing, bioimaging and LED devices.^21^

### Solvatochromism

Compound **5d** comprised of terminal NO_2_ group showed interesting solvatochromic property.^10^ Though the fluorescence emission of **5d** was quenched by some of the polar solvents, its emission was strong in apolar solvents. Specifically, 100 μM solutions of NO_2_-terminated analog **5d** in acetonitrile, acetone, THF, dioxane and chlorobenzene exhibits a strong solvatochromism. While the *λ*_max_ of the absorption spectra for the **5d** show a relatively small change (<20 nm) among all solvents, the corresponding emission spans the wavelengths from 480 to 570 nm, covering blue, green, yellow and orange region of visible spectrum (Fig. S5, ESI†).

Such strong solvatochromism occurs due to a combination of dipole–solvent interactions, intramolecular charge transfer, and aggregate formation in solution. The above result is also consistent with previously reported observations by our group and others where the conjugated oligomers possessing stronger acceptor end group(s) showing pronounced solvatochromism due to solvent stabilization of intramolecular charge transfer in the excited state.^10^

### pH and redox response

It is well established that perturbations in cellular redox status and pH have important physiological and pathological ramifications.^4,22^ Investigative tools to monitor these cellular responses are critical to further our understanding of the role of these processes in disease states. One important structural feature of these UAAs is the presence of the phenol group, which is particularly advantageous as it offers stimuli responsive properties in the following ways: (1) interchange between the phenol (acidic) and phenolate (basic) leads to a significant change in optical properties by varying the pH of the solution; and (2) when treated with an oxidizing agent, such as peroxide, the phenol undergoes oxidation to form the corresponding ketone, which causes perturbation in the conjugation length and thus effecting the optical properties. Since the optical responses of Tyr-OMPVs are reversible, these intrinsic properties were used to explore their potential application as pH and redox stimuli responsive sensors.

**Fig. 5:**
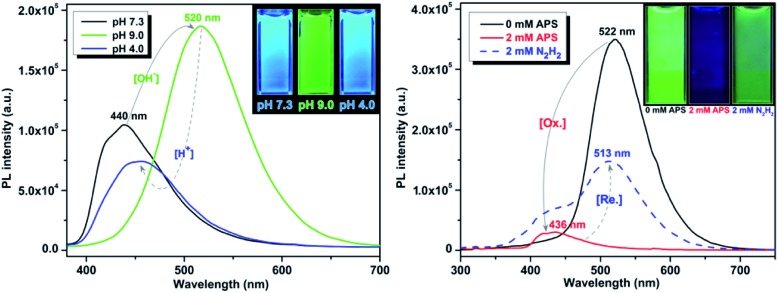
pH (left) and redox (right) sensitivity of compound **5b**. Emission spectra were recorded using 50 μM and 10 μM solutions, respectively.

A solution (DMSO–water 1 : 4 v/v) of **5b** was used to explore the stimuli responsive properties. As shown in (Fig. S6 to S8, ESI†) and Fig. 5, the absorption and emission spectrum of compound **5b** was sensitive to pH and redox stimuli. The absorption and emission spectra were recorded for this compound at two pH values (pH = 4.0 and 9.0). Emission of **5b** red shifted (from 440 nm to 520 nm) from pH 4 to 9, and its fluorescence intensity was much higher in the phenolate form than in the phenolic form. To establish the redox sensitivity, compound **5b** was subjected to an oxidation–reduction cycle in which ammonium persulfate (APS) was used as an oxidizing agent and hydrazine as a reducing agent. The addition of 2 mM of APS nearly completely quenched the fluorescence of **5b**, whereas the fluorescence was mostly recovered by the addition of 2 mM hydrazine. Note that fluorescence recovery was not 100% as the oxidized **5b** may have possibly reacted with moisture in the solution.

### NIR emission

Recently, Shabat and co-workers have reported on the design of NIR probes based on a D2A π-electron system that can undergo intramolecular charge transfer (ICT) to form a new fluorochrome with a longer π-conjugated system. In their design, phenol acts as a latent donor when it is conjugated with two acceptors. Deprotonation of the phenol transforms it into an active phenoxide donor, which donates a pair of π electrons to either one of the conjugated acceptors to exhibit ICT and thus emit at NIR region.^23^

As all of the molecules in this study were derived from tyrosine and contain a phenol ring, it is possible to design UAA chromophores with tunable and reversible optical properties wherein the emission extends into NIR by choosing the appropriate end group. Having a water-soluble NIR dye in the form of an amino acid could be useful for various imaging-related applications. Methylation of the pyridine moiety in compound **5f** gave a new analog **5g**, which comprises a phenol latent donor and two acceptors in the form of a pyridinium moiety.

**Fig. 6:**
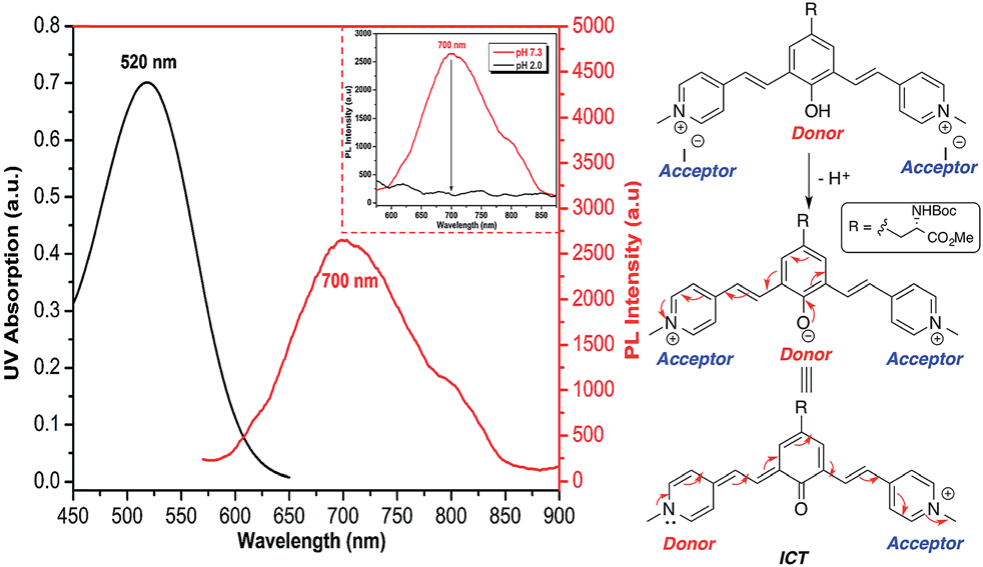
Absorption and emission spectra (left) of NIR emission of deprotonated **5g**; *c* = 100 μM dissolved in HEPES buffer (pH 7.3). Shown in inset is quenching of NIR emission upon acidification (pH 2) due to the protonation of phenolate; a schematic representation (right) showing the origin of NIR emission through ICT mechanism.

As described in Fig. 6, upon deprotonation of the phenol, an aqueous solution of compound **5g** showed an emission peak in the NIR region at a wavelength of 700 nm. It is known that the presence of the strong acceptor moieties in the dye decreases the p*K*_a_ of the phenol. Hence, the deprotonation of phenol of **5g** occurs under physiological pH and emits NIR fluorescence *via* ICT mode of action (Fig. S10, ESI†). Conversely, the NIR emission was diminished in acidic conditions (pH 2) due to the protonation of the phenolate. Fluorescence emission of **5g** in DMSO was further red-shifted to NIR region (800 nm), but the quantum yield decreased to 0.002, whereas it showed the moderate in context of NIR emission^24^ quantum yield (0.04) in aqueous solution.

### Utility in solid phase peptide synthesis (SPPS) and cell imaging

**Scheme 2:**
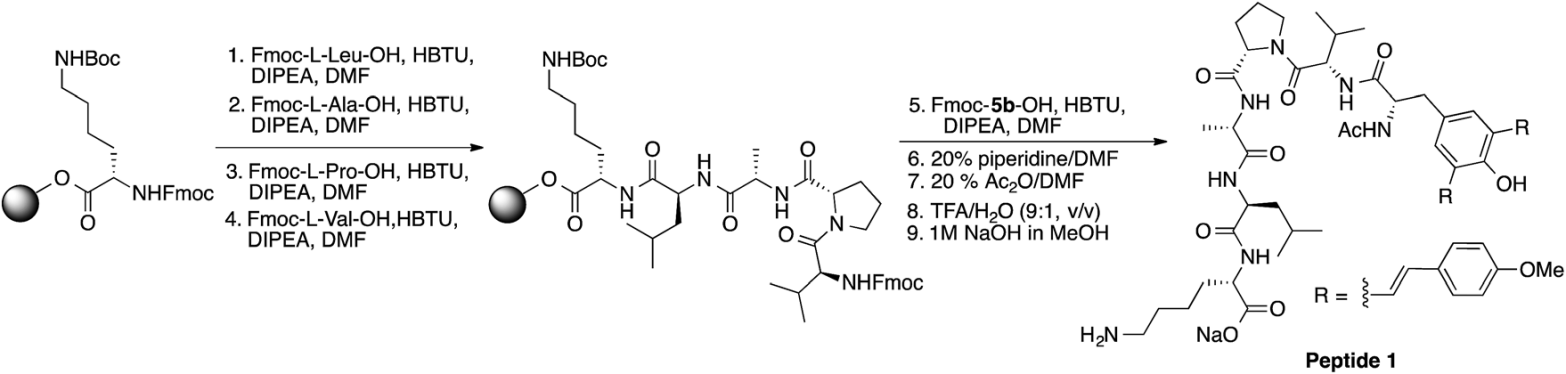
Solid phase synthesis of peptide 1.

In general, fluorescently labeled peptides are often obtained by the conjugation of the fluorescent dye through post-synthesis modifications. These typical bioconjugations often require fluorescent dye containing amine- or thiol reactive group which can be problematic in peptides bearing more than one amine or thiol group, presenting the possibility of generating either multiple labeled species or mixtures bearing different labeling patterns. In this regard, the use of fluorescent UAA **5b** allows for the direct and site-specific introduction of the fluorescent moiety as an integrated part of the SPPS process (Scheme 2, ESI†). In order to evaluate the utility of these amino acids in Fmoc-based peptide synthesis, an Fmoc analog of compound **5b** was synthesized and incorporated into a cell penetrating Bax-inhibiting pentapeptide^24^ (sequence = W*VPALK; W* = **5b**).

**Fig. 7:**
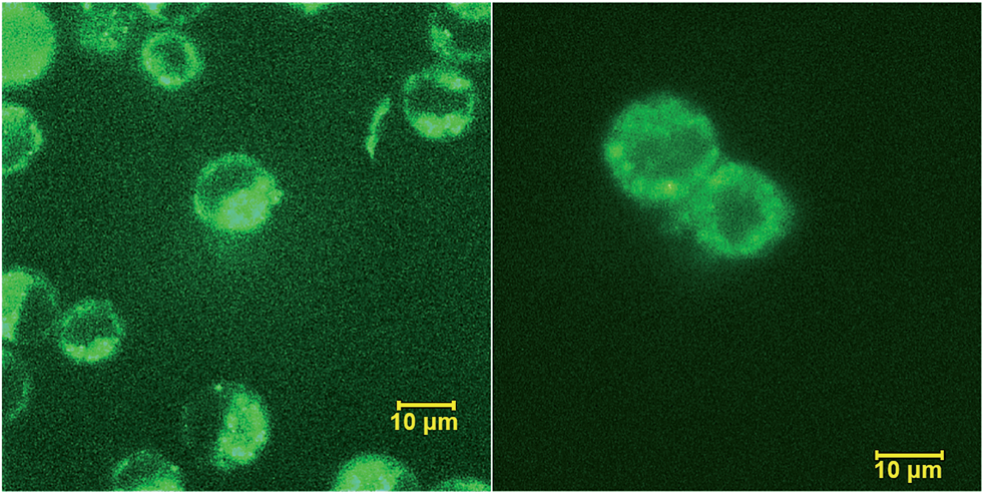
Internalization of peptide 1 by HeLa (left) and NIH 3T3 (right) cells visualized by confocal scanning microscope.

Peptide 1 was further characterized by UV-Vis and fluorescence spectroscopy over a range of pH values and the excitation and emission spectra are shown in Fig. S9, ESI.† To assess both the cell permeability and fluorescent properties, peptide 1 was incubated with a human epithelial cell line (HeLa) and mouse fibroblast cells (NIH 3T3) for 3 h and internalization was visualized using laser scanning confocal microscopy (Fig. 7), thus validating the use of these amino acids as intrinsic fluorescent labels.

## 3. Conclusions

In conclusion, we have developed a direct and facile synthesis of optically pure, fluorescent amino acids, using inexpensive natural amino acids as starting material avoiding expensive chiral auxiliaries and lengthy synthetic protocols. The combination of amino acid functionality and stimuli responsive *meta*-phenylene vinylene moiety in a molecule gives rise to a whole new class of fluorescent amino acids that display a broad range of optical properties extending into NIR region. Our synthetic platform utilizes simple Heck coupling reaction and represents a useful tool in the preparation of novel fluorescent amino acids, which can be easily incorporated into peptides and used in biological studies. The stimuli responsive nature of these UAAs is resulting from the change in the resonance structure upon variation of solution pH and redox states of the molecules; the UAA has a green color and higher quantum yield in high pH and reduced state, and exhibit blue color and lower quantum yield in low pH and oxidized state. Compound **5f** contains basic nitrogen atoms that can be protonated and a phenol group that can be deprotonated exhibits distinct red, green and blue (RGB) emission spectra simply by controlling the solution pH. Given the stimuli-responsive nature and the ability to emit NIR fluorescence, our UAAs exhibit a wide range of applications include fabricating optoelectronic devices, probing the cellular processes and other aspects of biological mechanism and function can be expected.
