# Supplementary material for: Tyrosine-derived stimuli responsive, fluorescent amino acids
Source: Chem Sci. 2014 Oct 31;6(2):1150–8. doi: 10.1039/c4sc02753a (PMC5811119; doi:10.1039/c4sc02753a)
Supplement: Supplementary file 1 [file SC-006-C4SC02753A-s001.pdf]

Electronic Supplementary Material (ESI) for Chemical Science.
This journal is © The Royal Society of Chemistry 2014

Supporting information

Tyrosine-Derived Stimuli Responsive, Fluorescent Amino Acids

Pradeep Cheruku,[a] Jen-Huang Huang,[b] Hung-Ju Yen,[a]
Rashi S. Iyer,[b] Kirk D. Rector,[a] Jennifer S. Martinez,[c] and Hsing-Lin Wang[a,*]

[a] C-PCS, Chemistry Division, [b] Defense System and Analysis Division, [c] Center of integrated Nanotechnologies (CINT), Los Alamos National Laboratory, Los Alamos, New Mexico, 87545, United States

*To whom correspondence should be addressed: **hwang@lanl.gov**

General experimental: All solvents and chemicals were purchased from Aldrich chemicals unless otherwise stated and were used without further purification. Fmoc protected amino acids, coupling reagents and resins were purchased from Novabiochem and used without further purification. Chromatographic separations were performed on Kiesel gel 60 H silica gel (particle size: 0.063-0.100 mm). Thin layer chromatography (TLC) was performed on aluminum-backed plates coated with Kieselgel 60 (0.20 mm, UV254) and neutral alumina visualized under ultraviolet light (ν = 254 nm), or by staining with ethanolic phosphomolybdic acid and heating. NMR spectra were recorded on Bruker spectrometers operating at 400 MHz for ^{1}H and 100 MHz for ^{13}C NMR. Chemical shifts are reported in ppm (δ scale) and coupling constants (J) are reported in Hz. Optical rotations were measured with a Jasco P-2000 polarimeter using a 1.0-dm cell.

General procedure for heck coupling: A mixture of the styrene (2.5 mmol), the Boc-3-iodo-L-tyrosine methyl ester or Boc-3,5-diiodo-L-tyrosine (1 mmol), DIPEA (5 mmol), $Pd(OAc)_2$ (5 mol%) and P(*o*-tol)$_3$ (7 mol%) in anhydrous DMF was stirred under nitrogen at 100 °C for 4-12 h. The reaction was cooled to 25 °C and filtered through celite. The filtrate was collected and evaporated in vacuum. The crude product was purified by column chromatography.

Methyl(*R*,*E*)-2-((*tert*-butoxycarbonyl)amino)-3-(4-hydroxy-3-styrylphenyl)propanoate(4a):
(corresponding carboxylic acid analog)

Chromatographed on silica gel (n-hexane/ethyl acetate) to obtain **4a** as light brown solid, yield = 81%; $[\alpha]_D^{25}$: +15 (c = 0.08, methanol); ^{1}H NMR (400 MHz, DMSO-*d$_6$*): δ 9.50 (s, 1H), 7.60-7.49 (m, 2H), 7.48-7.31 (s, 2H), 7.30-7.20 (m, 1H), 7.16 (d, *J* = 16.80 Hz, 1H), 7.09-6.93 (m, 1H), 6.90-6.82 (m, 1H), 6.83-6.73 (m, 1H), 4.21-4.09 (m, 1H), 2.94-2.85 (m, 1H), 2.80-2.70 (m, 1H), 1.30 (s, 9H); ^{13}C NMR (75 MHz, $CDCl_3$): δ 171.60, 154.20, 152.71, 139.09, 137.79, 131.04, 129.97, 129.70, 128.61, 127.84, 127.45, 126.57, 123.22, 116.38, 115.17, 85.34, 80.68, 60.60, 28.20; MS (MALDI-TOF): calcd for: $C_{23}H_{27}NO_5$ = 397.2, observed = 397.1

Methyl(*R*,*E*)-2-((*tert*-butoxycarbonyl)amino)-3-(4-hydroxy-3-(4-methoxystyryl)phenyl)propanoate (4b): Chromatographed on silica gel in n-hexane/ethyl acetate system to obtain **4b** as pale yellow solid, yield = 72 %; $[\alpha]_D^{25}$: +16 (c = 0.1, methanol); ^{1}H NMR (400 MHz, DMSO-*d$_6$*): δ 9.53 (s, 1H), 7.47 (d, *J* = 7.46, 2H), 7.41 (s, 1H), 7.29-7.18 (m, 2H), 7.10 (d, *J* = 16.65 Hz, 1H), 6.98-6.87 (m, 3H), 6.75 (d, *J* = 8.39 Hz, 1H), 4.21-4.09 (m, 1H), 3.77 (s, 3H), 3.62 (s, 3H), 2.95-2.86 (m, 1H), 2.80-2.70 (m, 1H), 1.30 (s, 9H); ^{13}C NMR (75 MHz, $CDCl_3$): δ 172.76, 159.19, 155.37, 152.59, 130.57, 130.08, 129.13, 128.81, 127.74, 127.48, 124.98, 121.03, 116.12, 114.11, 113.94, 80.26, 55.32, 54.67, 52.34, 37.71, 28.33; MS (MALDI-TOF): calcd for: $C_{24}H_{29}NO_6$ = 427.2, observed = 427.1

Methyl (*R*,*E*)-3-(3-(4-aminostyryl)-4-hydroxyphenyl)-2-((*tert*-butoxycarbonyl)amino)propanoate (4c): A suspension of compound **4d** and catalytic amount of Rany-Ni in ethyl acetate/methanol (1:1 v/v) was added hydrazine drop wise and stirred at room temperature until the reaction color turned to green. At this point, some more hydrazine was added and stirred for 10 more min. The reaction mixture was filtered through cilite and washed the celite bed with 1:1 ethyl acetate/methanol. Filtrate and washings were

collected and evaporated in vacuo. The obtained solid was purified by column chromatography (n-hexane/ethyl acetate) to yield compound **4c** as yellow solid in 87 % yield**.** $[\alpha]_D^{25}$: +11 (c = 0.08, methanol); ^{1}H NMR (400 MHz, DMSO-d_6): δ 7.38 (s, br, 2H), 7.21 (d, *J* = 8.26 Hz, 1H), 7.00 (d, *J* = 16.9 Hz, 1H), 6.90-6.78 (m, 3H), 6.75-6.71(m, 2H), 6.57 (d, *J* = 8.24 Hz, 2H), 4.21-3.80 (m, br, 1H), 4.50-3.70 (m, br, 3H), 3.40 (s, 3H), 2.95-2.86 (m, 1H), 2.80-2.70 (m, 1H), 1.30 (s, 9H); ^{13}C NMR (75 MHz, DMSO-d_6): δ 170.00, 155.60, 153.32, 152.59, 148.90, 130.54, 128.98, 128.70, 128.56, 127.66, 126.67, 126.06, 124.70, 118.67, 115.77, 114.51, 78.39, 55.22, 37.78, 28.62; MS (MALDI-TOF): calcd for: $C_{23}H_{28}N_2O_5$ = 412.2, observed = 412.3

Methyl (*R*,*E*)-2-((*tert*-butoxycarbonyl)amino)-3-(4-hydroxy-3-(4-nitrostyryl)phenyl)propanoate (4d): Chromatographed on silica gel in n-hexane/ethyl acetate system followed by the crystallization in CH_2Cl_2 to obtain **4d** as orange-yellow solid, yield = 72 %; $[\alpha]_D^{25}$: +10 (c = 0.13, methanol); ^{1}H NMR (400 MHz, DMSO-d_6): δ 9.85 (s, 1H), 8.22 (d, *J* = 8.90, 2H), 7.80 (d, *J* = 8.90, 2H), 7.63 (d, *J* = 16.56 Hz, 1H), 7.52 (s, 1H), 7.35 (d, *J* = 8.90, 2H), 7.27 (d, *J* = 8.03 Hz, 1H), 7.07-7.00 (m, 1H), 4.22-4.10 (m, 1H), 3.62 (s, 3H), 2.98-2.86 (m, 1H), 2.84-2.72 (m, 1H), 1.32 (s, 9H); ^{13}C NMR (75 MHz, $CDCl_3$): δ 173.15, 155.85, 154.80, 146.40, 145.19, 131.13, 129.09, 128.67, 128.10, 127.40, 126.06, 124.59, 123.09, 116.35, 78.73, 55.87, 52.18, 36.27, 28.62; MS (ESI): calcd for: $C_{23}H_{26}N_2O_7$ = 442.2, observed = 442.3

Methyl (*R*,*E*)-2-((*tert*-butoxycarbonyl)amino)-3-(4-hydroxy-3-(4-(trifluoromethyl)styryl)phenyl) propanoate (4e): Chromatographed on silica gel in n-hexane/ethyl acetate system to obtain **4e** as pale yellow solid, yield = 86 %; $[\alpha]_D^{25}$: +15 (c = 0.1, methanol); ^{1}H NMR (400 MHz, DMSO-d_6): δ 9.80 (s, 1H), 8.55-8.49 (m, 2H), 7.64 (d, *J* = 16.56, 1H), 7.54-7.43 (m, 3H), 7.27 (d, *J* = 8.16 Hz 2H), 7.18 (d, *J* = 16.65 Hz, 1H), 7.06-6.99 (m, 1H), 6.80 (d, *J* = 8.27 Hz, 1H), 4.23-4.11 (m, 1H), 3.62 (s, 3H), 3.01-2.83 (m, 1H), 2.84-2.69 (m, 1H), 1.32 (s, 9H); ^{13}C NMR (75 MHz, $CDCl_3$): δ 173.14, 155.86, 154.74, 150.49, 150.07, 145.36, 130.98, 130.61, 130.25, 128.80, 128.20, 127.19, 125.66, 122.97, 121.05, 116.31, 78.75, 55.93, 36.31, 31.16, 28.63; MS (ESI): calcd for: $C_{24}H_{26}F_3NO_5$= 465.2, observed = 465.2

Methyl(*R*,*E*)-2-((*tert*-butoxycarbonyl)amino)-3-(4-hydroxy-3-(2-(pyridin-4-yl)vinyl)phenyl) propanoate (4f): Chromatographed on silica gel in n-hexane/ethyl acetate system to obtain 4 as pale yellow solid, yield = 72 %; $[\alpha]_D^{25}$: +12 (c = 0.1, methanol); ^{1}H NMR (400 MHz, DMSO-d_6): δ 9.81 (s, 1H), 8.56-8.55 (m, 2H), 7.64 (d, *J* = 16.59, 1H), 7.54-7.44 (m, 3H), 7.27 (d, *J* = 8.13 Hz, 1H), 7.18 (d, *J* = 16.59 Hz, 1H), 7.06-6.99 (m, 1H), 6.80 (d, *J* = 8.13 Hz, 1H), 4.22-4.10 (m, 1H), 3.62 (s, 3H), 2.99-2.84 (m, 1H), 2.84-2.69 (m, 1H), 1.32 (s, 9H); ^{13}C NMR (75 MHz, $CDCl_3$): δ 173.13, 155.87, 154.73, 150.50, 145.37, 130.98, 128.82, 128.62, 128.21, 125.69, 122.98, 121.06, 116.34, 78.74, 55.86, 52.19, 36.28, 28.61; MS (ESI): calcd for: $C_{22}H_{26}N_2O_5$ = 398.2, observed = 398.4

Methyl (*R*)-2-((*tert*-butoxycarbonyl)amino)-3-(4-hydroxy-3,5-di((*E*) styryl) phenyl) propanoate (5a): Chromatographed on silica gel in n-hexane/ethyl acetate system to obtain **5a** as pale yellow foam, yield = 70%. $[\alpha]_D^{25}$: +8 (c = 0.1, methanol); ^{1}H NMR (400 MHz, DMSO-d_6): δ 7.57-7.49 (d, *J* = 8.75, 4H), 7.41 (s, 2H), 7.40 (d, *J* = 16.20, 2H), 7.35 (d, *J* = 8.05, 1H), 7.09 (d, *J* = 16.20, 2H), 6.96 (d, *J* = 8.75, 5H), 4.22 (m, 1H), 3.78 (s, 6H), 3.65 (s, 3H), 3.03-2.63 (m, 2H), 1.30 (s, 9H); ^{13}C NMR (75 MHz, $CDCl_3$): δ 172.53,

155.42, 150.26, 148.21, 137.66, 137.62, 131.28, 130.89, 129.09, 128.98, 128.51, 128.46, 128.12, 128.09, 127.37, 126.97, 126.89, 126.57, 126.09, 125.83, 123.14, 122.82, 120.76, 80.39, 77.56, 54.82, 52.64, 52.60, 37.90, 28.63; MS (MALDI-TOF): calcd for: $C_{31}H_{33}NO_5$ $[M + H]^+$ = 499.2, observed = 499.1

Methyl(*R*)-2-((*tert*-butoxycarbonyl)amino)-3-(4-hydroxy-3,5-bis((*E*)-4-methoxystyryl)phenyl) propanoate (5b): Chromatographed on silica gel in n-hexane/ethyl acetate system to obtain **5b** as pale yellow solid, yield = 52 %; $[\alpha]_D^{25}$: +10 (c = 0.1, methanol); ^{1}H NMR (400 MHz, DMSO-d_6): δ 12.55 (br, 1H), 8.96 (br, 1H), 7.53 (d, *J* = 8.75 Hz, 4H), 7.41 (s, 2H), 7.40 (d, *J* = 16.40, 2H), 7.13 (d, *J* = 8.37, 1H), 7.08 (d, *J* = 16.40, 2H), 6.97 (d, *J* = 8.75, 4H), 4.14 (m, 1H), 3.78 (s, 6H), 3.06-2.69 (m, 2H), 1.32 (s, 9H) (corresponding carboxylic acid analog); ^{13}C NMR (75 MHz, $CDCl_3$): δ 172.59, 159.45, 149.55, 130.66, 135.50, 130.39, 130.16, 129.74, 129.23, 128.10, 127.81, 127.82, 127.74, 126.36, 125.68, 120.65, 114.15, 114.09, 80.03, 55.33, 54.65, 52.30, 37.79, 28.33; MS (MALDI-TOF): calcd for: $C_{32}H_{35}NO_7$ = 545.2, observed = 545.1

Methyl(*R*)-3-(3,5-bis((*E*)-4-aminostyryl)-4-hydroxyphenyl)-2-((*tert*-butoxycarbonyl)amino) propanoate(5c): A suspension of compound **5d** and catalytic amount of Rany-Ni in ethyl acetate/methanol (1:1 v/v) was added hydrazine drop wise and stirred at room temperature until the reaction color turned to green. At this point, some more hydrazine was added and stirred for 10 more min. The reaction mixture was filtered through cilite and washed the celite bed with 1:1 ethyl acetate/methanol. Filtrate and washings were collected and evaporated in vacuo. The obtained solid was purified by column chromatography (n-hexane/ethyl acetate) to yield compound **5c** as yellowish green solid in 80 % yield**.** $[\alpha]_D^{25}$: +8 (c = 0.1, methanol); ^{1}H NMR (400 MHz, DMSO-d_6): δ 8.75 (s, 1H), 7.55-7.10 (m, 10H), 6.93 (d, *J* = 16.20, 2H), 6.56 (d, *J* = 8.17, 4H), 5.32 (br, 4H), 4.31-4.13 (m, 1H), 3.65 (s, 6H), 3.05-2.86 (m, 1H), 2.85-2.68 (m, 1H), 1.30 (s, 9H); ^{13}C NMR (75 MHz, DMSO-d_6): δ 172.30, 155.87, 150.00, 148.96, 129.22, 129.15, 127.91, 126.97, 126.68, 125.85, 124.63, 118.14, 114.40, 78.70, 55.86, 52.25, 31.18, 28.59; MS (MALDI-TOF): calcd for: $C_{31}H_{35}N_3O_5$ = 529.2, observed = 529.1

Methyl (*R*)-2-((*tert*-butoxycarbonyl)amino)-3-(4-hydroxy-3,5-bis((*E*)-4-nitrostyryl)phenyl)propanoate (5d): Chromatographed on silica gel in dichloromethane/methanol system and precipitated in dichloromethane to obtain **5d** as orange solid solid, yield = 40%, $[\alpha]_D^{25}$: +12 (c = 0.1, methanol); ^{1}H NMR (400 MHz, DMSO-d_6): δ 9.60 (s, 1H), 8.27 (d, *J* = 8.75, 4H), 7.85 (d, *J* = 8.75, 4H), 7.81 (d, *J* = 16.57, 2H), 7.62 (s, 2H), 7.38 (d, *J* = 8.02, 1H), 7.34 (d, *J* = 16.37, 2H), 4.32-4.22 (m, 1H), 3.65 (s, 3H), 3.05-2.97 (m, 1H), 2.88-2.79 (m, 1H), 1.30 (s, 9H); ^{13}C NMR (75 MHz, DMSO-d_6): δ 173.12, 155.88, 152.39, 146.54, 144.92, 129.65, 128.66, 128.16, 127.60, 126.81, 125.35, 124.59, 78.73, 55.67, 52.27, 36.36, 28.56; MS (ESI): calcd for: $C_{31}H_{31}KN_3O_9$ = 629.2, observed = $[M + H+K]^+$ = 629.4

Sodium 4,4'-((1*E*,1'*E*)-(5-((*R*)-2-((*tert*-butoxycarbonyl)amino)-3-methoxy-3-oxo propyl)-2-hydroxy-1,3-phenylene)bis(ethene-2,1-diyl))dibenzenesulfonate(5e): After the reaction, solvents were evaporated and the obtained solid was re-dissolved in small amount of DMF and added to 1:1 acetone-methanol mixture. The precipitated was filtered and the obtained solid was washed with 1:10 water/methanol mixture. Obtained as a mixture of **5e** and the starting material 4-vinylbenzenesulfonate. 10 mg of crude

mixture was further purified by size exclusion chromatography (SEC) using Bio-Gel P-2 gel purchased from Bio-Rad to obtain relatively pure **5e** (purity between 85-90% confirmed by ^{1}H NMR) as off white powder (whitish fluffy solid after lyophilization). $[\alpha]_D^{25}$: +15 (c = 0.1, water), ^{1}H NMR (400 MHz, DMSO-*d*$_6$): δ 8.20 (d, *J* = 8.40, 4H), 7.86 (d, *J* = 16.43, 2H), 7.79 (d, *J* = 8.40, 4H), 7.55 (s, 2H), 7.38 (d, *J* = 8.02, 1H), 7.33 (d, *J* = 16.43, 2H), 4.38-4.22 (m, 1H), 3.64 (s, 3H), 3.15-2.97 (m, 1H), 2.90-2.76 (m, 1H), 1.30 (s, 9H); ^{13}C NMR (75 MHz, DMSO-*d*$_6$): δ 172.60, 155.45, 150.32, 148.22, 137.76, 137.65, 131.38, 130.92, 129.19, 129.09, 128.55, 128.56, 128.22, 128.21, 127.47, 127.01, 126.87, 126.77, 126.19, 126.01, 123.14, 123.02, 120.76, 80.39, 77.56, 54.82, 52.64, 52.72, 38.00, 28.73; MS (ESI): calcd for: $C_{30}H_{30}NNaO_{11}S$ = 667.1, observed = $[M + H]^+$ = 668.1 (corresponding carboxylic acid)

Methyl(*R*)-2-((*tert*-butoxycarbonyl)amino)-3-(4-hydroxy-3,5-bis((*E*)-2-(pyridin-4-yl)vinyl)phenyl) propanoate(5f): Chromatographed on neutral alumina in dichloromethane/methanol system to obtain **5f** as a yellow solid, yield = 35 %, $[\alpha]_D^{25}$: +12 (c = 0.23, methanol); ^{1}H NMR (400 MHz, DMSO-*d*$_6$): δ 9.50 (s, 1H), 8.57 (m, 4H), 7.82 (d, *J* = 16.43, 4H), 7.58 (s, 2H), 7.54 (m, 4H), 7.36 (d, *J* = 16.43, 2H), 7.17 (d, *J* = 16.43 Hz, 2H), 4.30-4.20 (m, 1H), 3.65 (s, 3H), 3.06-2.96 (m, 1H), 1.30 (s, 9H); ^{13}C NMR (75 MHz, DMSO-*d*$_6$): δ 173.16, 155.88, 152.15, 145.11, 129.64, 128.38, 128.16, 126.39, 125.18, 121.26, 78.75, 55.67, 52.32, 36.29, 28.53; MS (MALDI-TOF): calcd for: $C_{29}H_{31}N_3O_5$ = 501.2, observed $[M + H]^+$ = 502.1

4,4'-((1*E*,1'*E*)-(5-((*R*)-2-((*tert*-butoxycarbonyl)amino)-3-methoxy-3-oxopropyl)-2-hydroxy-1,3 phenylene)bis(ethene-2,1-diyl))bis(1-methylpyridin-1-ium)iodide(5g): Compound **5f** (1eqiv) was dissolved in anhydrous acetone and added iodomethane (5 equiv) and stirred at room temperature for 1 h. The reaction mixture was heated to 40 ^{o}C for additional 3 h and the solvents were removed under reduced pressure. The resulted solid was precipitated in cold diethyl ether to obtain **5g** as dark brown solid in a quantitative yield. $[\alpha]_D^{25}$: +10 (c = 0.01, water); ^{1}H NMR (400 MHz, DMSO-*d*$_6$): δ 8.84 (d, *J* = 6.43, 4H), 8.27-8.12 (m, 6H), 7.96 (s, 1H), 7.73 (s, 2H), 7.48 (d, *J* = 16.08, 2H), 7.34 (d, *J* = 8.04, 1H), 4.27 (m, 6H), 3.65 (s, 3H), 3.09-2.97 (m, 2H), 1.30 (s, 9H); ^{13}C NMR (75 MHz, DMSO-*d*$_6$): δ 173.04, 155.90, 155.85, 152.97, 145.65, 135.71, 130.79, 124.53, 123.98, 123.92, 78.84, 52.36, 50.54, 47.41, 34.83, 28.53; MS (ESI): calcd for: $C_{31}H_{37}N_3O_5$ $[M + H]^+$ = 532.3, obsd $[M + H]^+$ = 532.2

Peptide Synthesis:

General method for SSPS :[1] Sequence [2]: W*VPALK (W* = **5b**)

Solid-phase peptide synthesis was carried out manually on 0.01mmol scale in a solid phase peptide synthesis vessel having a medium or coarse porosity fritted glass support.

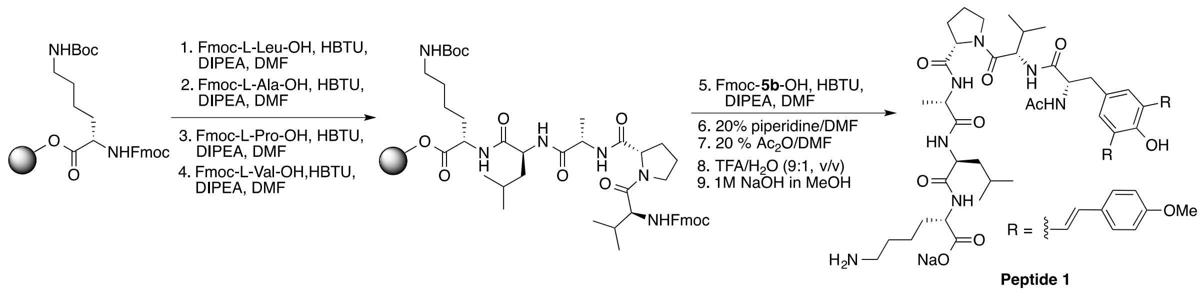

Scheme S1: Solid Phase Synthesis of Peptide 1

The Fmoc-Val-Wang resin was subjected to Fmoc-peptide synthesis using the following conditions:

Swelling: Fmoc-Lys(Boc)-Wang resin (100-200 mesh), 0.61 meq, 0.016 g, 0.01 mmol) was immersed in DCM (10 mL) and swelled for 2h. Drained and washed with DMF (2 x 5 mL)

Fmoc removal: The resin was treated with a solution of 20% piperidine/DMF (5 mL) for 30 min, and then washed with DMF (5 x 5 mL), dichloromethane (5 x 5mL) and DMF (5 x 5 mL).

Capping: The resin was treated with a solution of 20% acetic anhydride/DMF (10 mL) for 30 min and washed with DMF (5 x 5 mL) and dichloromethane (5 x 5mL).

Coupling conditions: An appropriate Fmoc-protected amino acid (3 equiv), HBTU (3 equiv) and DIPEA (8 equiv) in 5 mL DMF was allowed to stand for 10 min, and the resulting solution was added to the resin and agitated for 3 h. The resin was drained and washed with DMF (5 x 5 mL) and dichloromethane (5 x 5 mL) and DMF (5 x 5 mL). When HBTU was used as a coupling reagent to incorporate Fmoc protected **3b** into the peptide in its unprotected phenol form, the reaction did not provide the desired peptide in good yield. Therefore, we chose 3-(diethoxyphosphoryloxy)-1,2,3-benzotriazin-4(3*H*)-one (DEPBT)[(ref)] as a coupling reagent as it is known to mediate amide bond formation without protecting to the hydroxyl group of the amino acids such as serine and tyrosine. Using DEPBT, coupling went smooth obtaining the final peptides in good yields.

Release: The product was cleaved from the solid support by treatment with TFA:H_2O (9:1, v/v, 10 mL) for 3 h. The resin was filtered and washed with TFA (2 x 5 mL) and dichlorormethane (2 x 5 mL). The combined filtrate and washings were removed in vacuo to give the crude peptide, which was dissolved in small amount of TFA and precipitated with *tert*-butyl ether. The precipitate was dissolved in ACN/H_2O (1:1) and lyophilized to obtain relatively pure peptide in 60% overall yield which was further purified by RP HPLC.

HPLC analysis: Waters C18, 5 µm LC Column100×2mm, 220 and 300 nm (t=0−5min, 90% A, 10% B; t=5−60min, 10% A, 90% B, t=61−70min, 100% B. (A= water, 0.05% TFA, B = methanol). Flow rate = 0.5 mL/min, tR = 45.89 min); MS (ESI): calcd for: $C_{54}H_{72}N_7NaO_{11}$ = 1017.5, observed = $[M + H]^+$ = 1018.3

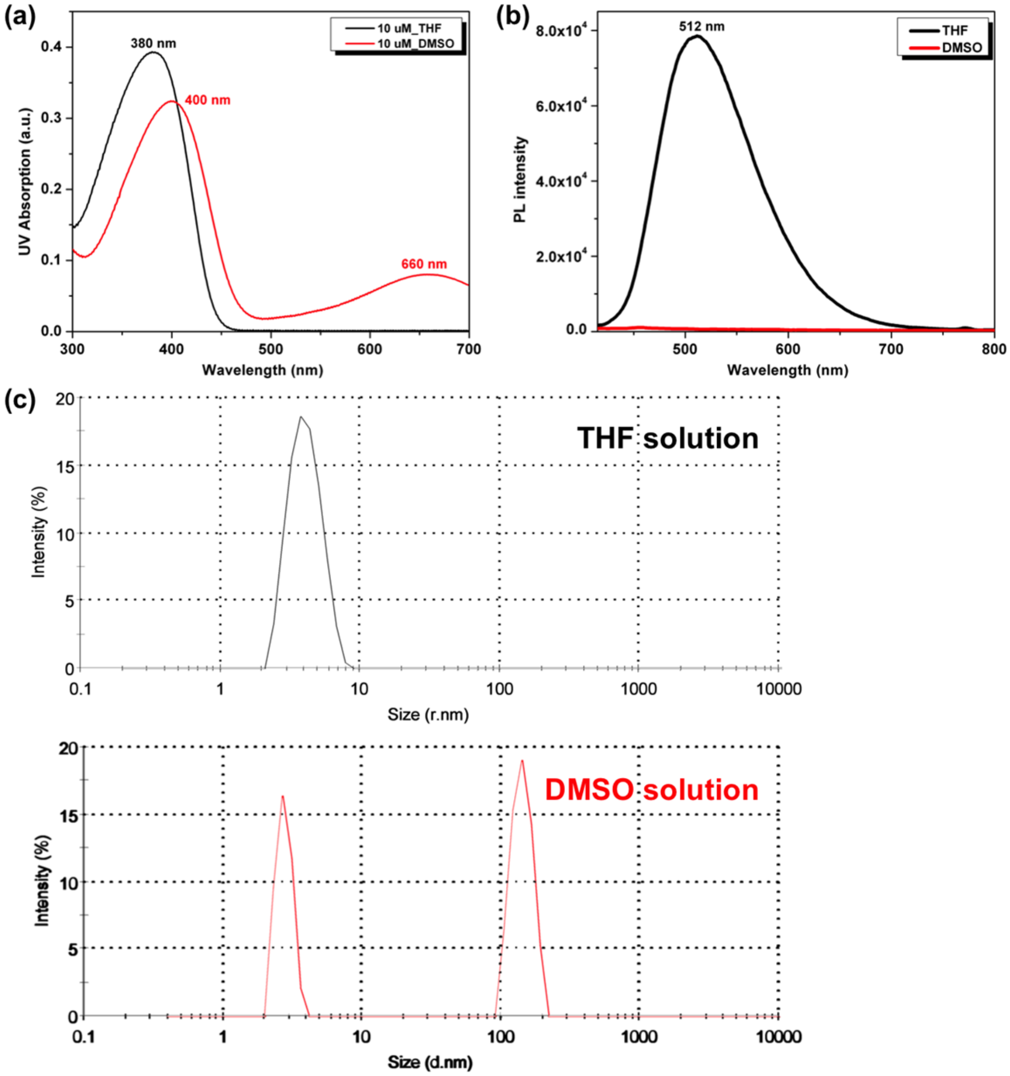

Figure S1: (a) Absorption, (b) emission spectra, and (c) DLS data of compound **5d**: comparison in THF and DMSO

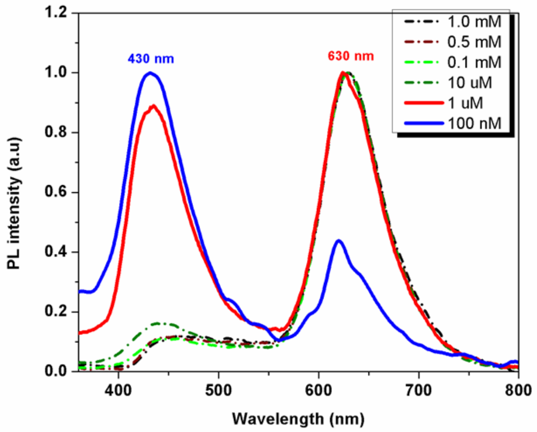

Figure S2: Concentration dependent PL spectra of pyridine analog **5f** in DMSO showing the aggregate formation at concentrations higher than 100 nM

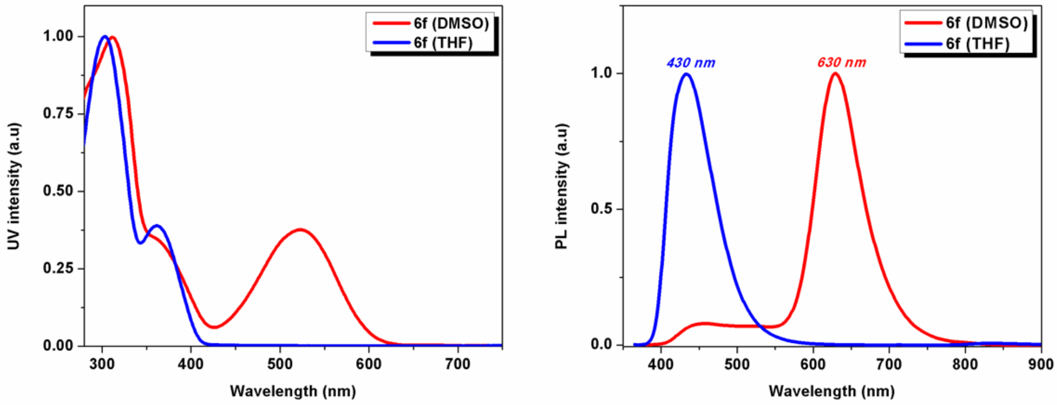

Figure S3. Comparison of UV-vis and PL spectra of pyridine analog **(5f)** in DMSO and THF

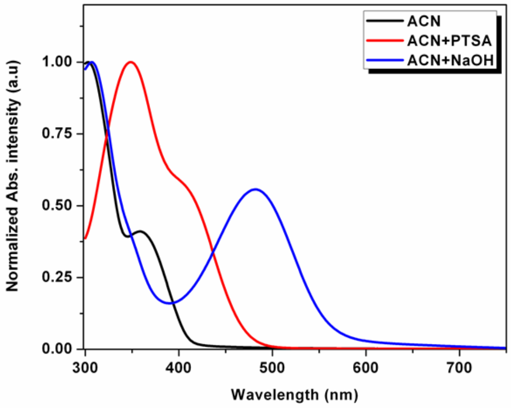

Figure S4. UV-vis spectra of pyridine analog **(5f)** in acetonitrile under neutral, acidic and basic environments

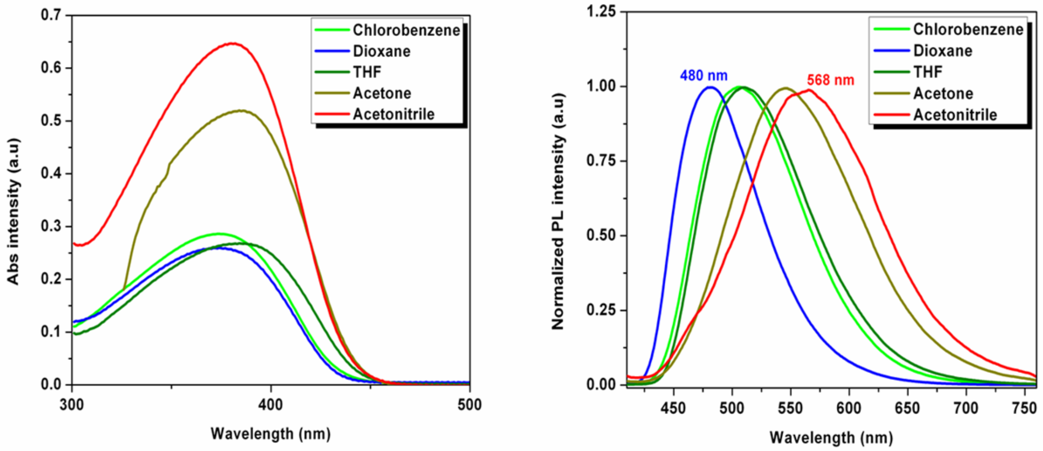

Figure S5. UV-vis and PL spectra of NO_2 analog **(5d)** in different solvents (solvatochromism)

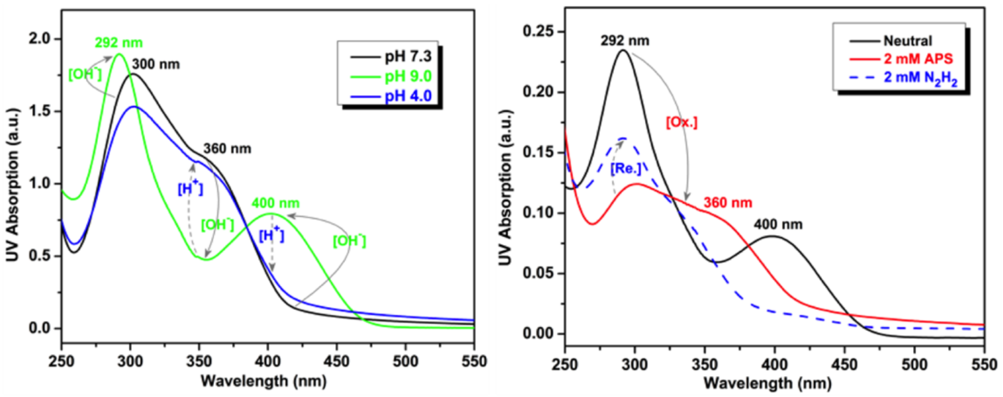

Figure S6. pH (left) and redox (right) sensitivity of the compound **5b.** UV-vis spectra were recorded using 50 µM and 10 µM solutions respectively

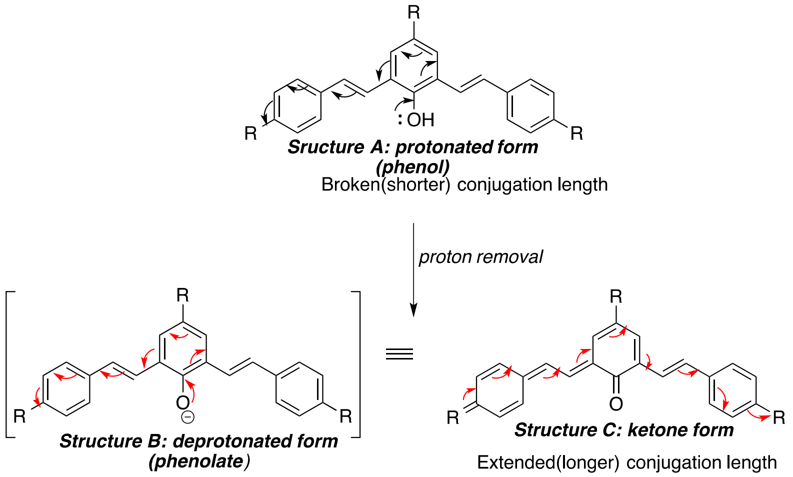

Figure S7: Origin of pH sensitivity for **5b:** Structure A (pH 4) is responsible for the blue emission color under acidic conditions whereas structure B and C (pH 9) showed a redshift in emission spectrum due to the extended conjugation and thus responsible for the green emission color

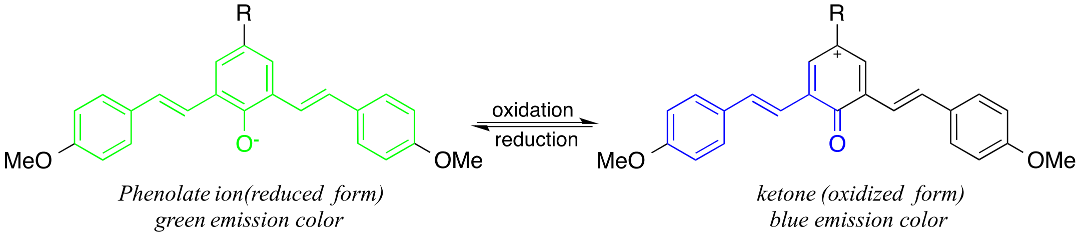

Figure S8: Origin of redox sensitivity for compound **5b**

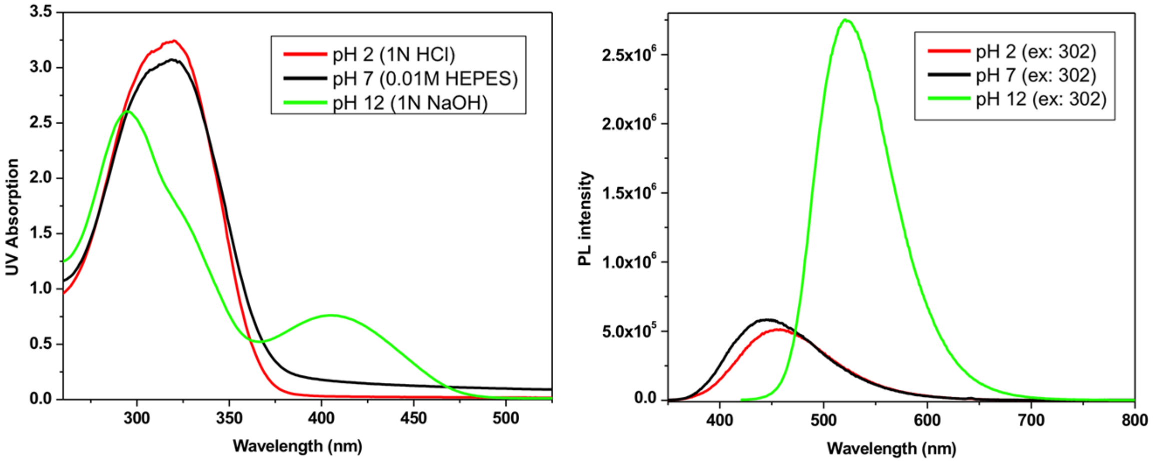

Figure S9: Absorption (left) and emission (right) spectra recorded at pH 2, 7, and 12 for peptide 1

Peptide 1 showed a clear pH dependence of the fluorescence. While the fluorescence spectra for peptide 1 at both pH 2 and 7 are essentially identical, emission color at pH 12 is different. The optical spectra obtained at pH 12 showed an additional peak at 420 nm in UV-vis spectrum and a red-shifted emission maximum, which are attributed to the deprotonated (phenoxide) form of the phenol of the amino acid.

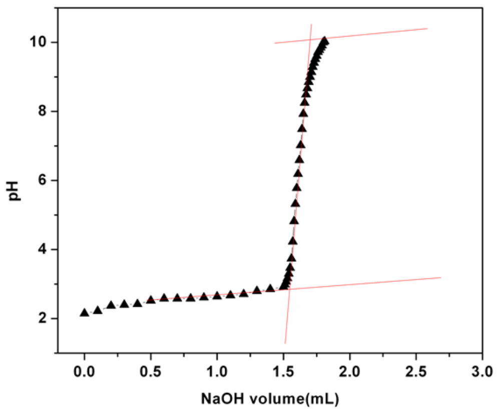

Figure S10: Determination of pKa value for phenol of **5g**; titration curve

References:

1. Cheruku, P.; Plaza, A.; Lauro, G.; Keffer, J.; Lloyd, J.; Bifulco, G.; Bewley, A. J. Med. Chem. 2012, 55, 735
2. Gomez, J. A.; Chen, J.; Ngo, J.; Hajkova, D.; Yeh, I.-J.; Gama, V.; Miyagi, M.; Matsuyama, S. *Pharmaceuticals,* **2010**, *3*, 3594
